# Neural Stem Cells Achieve and Maintain Pluripotency without Feeder Cells

**DOI:** 10.1371/journal.pone.0021367

**Published:** 2011-06-24

**Authors:** Hyun Woo Choi, Jong Soo Kim, Sol Choi, Hyo Jin Jang, Min Jung Kim, Youngsok Choi, Hans R. Schöler, Hyung Min Chung, Jeong Tae Do

**Affiliations:** 1 Department of Biomedical Science, Stem Cell and Developmental Biology, CHA University, Seoul, Republic of Korea; 2 CHA Bio and Diotech, Seoul, Republic of Korea; 3 Department of Biomedical Science, CHA University, Seoul, Republic of Korea; 4 Department of Cell and Developmental Biology, Max Planck Institute for Molecular Biomedicine, Münster, Germany; Center for Regenerative Therapies Dresden, Germany

## Abstract

**Background:**

Differentiated cells can be reprogrammed into pluripotency by transduction of four defined transcription factors. Induced pluripotent stem cells (iPS cells) are expected to be useful for regenerative medicine as well as basic research. Recently, the report showed that mouse embryonic fibroblasts (MEF) cells are not essential for reprogramming. However, in using fibroblasts as donor cells for reprogramming, individual fibroblasts that had failed to reprogram could function as feeder cells.

**Methodology/Principal Finding:**

Here, we show that adult mouse neural stem cells (NSCs), which are not functional feeder cells, can be reprogrammed into iPS cells using defined four factors (Oct4, Sox2, Klf4, and c-Myc) under feeder-free conditions. The iPS cells, generated from NSCs expressing the *Oct4*-GFP reporter gene, could proliferate for more than two months (passage 20). Generated and maintained without feeder cells, these iPS cells expressed pluripotency markers (*Oct4* and *Nanog*), the promoter regions of *Oct4* and *Nanog* were hypomethylated, could differentiated into to all three germ layers in vitro, and formed a germline chimera. These data indicate that NSCs can achieve and maintain pluripotency under feeder-free conditions.

**Conclusion/Significance:**

This study suggested that factors secreted by feeder cells are not essential in the initial/early stages of reprogramming and for pluripotency maintenance. This technology might be useful for a human system, as a feeder-free reprogramming system may help generate iPS cells of a clinical grade for tissue or organ regeneration.

## Introduction

Pluripotent embryonic stem (ES) cells not only have potential to differentiate into all three germ layers but also ability to self-renew for an expanded period of time [1]. These capabilities of pluripotent stem cells are expected to be useful for regenerative medicine as well as basic research [2], [3]. However, ethical issues have shackled the clinical applications of ES cells. Yamanaka and colleagues showed that a noble source of pluripotent stem cells could be directly derived from differentiated somatic cells by transduction of reprogramming factors [4]. The induced pluripotent stem (iPS) cells were generated by transduction of four defined transcription factors (*Oct4, Sox2, Klf4,* and *c-Myc*) from mouse and human somatic cells [4]–[7]. These iPS cells were indistinguishable from ES cells in expression of pluripotency-associated genes and differentiation potential *in vitro* and *in vivo* [8]–[11].

Mouse embryonic fibroblasts (MEFs) have been used as a feeder cells to support ES cell derivation and iPS cell generation. Pluripotent ES cells have been derived from the inner cell mass (ICM) of blastocysts and can be expanded *in vitro* in a culture with inactivated MEF feeder cells [12]–[14]. Differentiated cells have been reprogrammed on MEF feeder cells, which provide a reprogramming microenvironment for generating iPS cells. However, recent reports indicate that the iPS cells were induced to differentiate when cultured in the absence of feeder cells [4], [7], suggesting that MEF feeder cells provide a supportive reprogramming environment [4], [9]. MEF feeder cells secret multiple proteins and soluble factors, such as Activin A, TGF ß, WNT, and BMP4, which are important in maintaining proliferation and pluripotency of ES cells [15], [16]. However, MEF and human fibroblast feeder cells are inactivated by irradiation and mitomycin C, treatment that may cause DNA damage to pluripotent stem cells and the chemical of which may persist in the culture system after extensive washing [17].

Therefore, future application involving iPS cell–derived cells or tissues in human is contingent upon the ability of a feeder–free culture system to support the long-term stability of cell lines. Thus, many researchers have attempted to induce and maintain pluripotency of iPS cells in a feeder-free culture system in mouse and human [18]–[23]. Recently, Chen et al. showed that MEF cells were not necessary for the initial step of reprogramming, but were important for maintaining iPS cell proliferation [20]. However, in the reprogramming culture, individual fibroblasts that had failed to reprogram could function as feeder cells [24] and support reprogrammed cells. Those authors did not verify whether the iPS cells cultured in the feeder-free condition are functionally pluripotent, either by showing chimera formation after blastocyst injection or by aggregation methods.

In this study, we demonstrated that a feeder cell is not essential for reprogramming somatic cells into iPS cells and maintaining iPS cell pluripotency. To investigate whether iPS cells could be generated and maintained without feeder cells, we generated iPS cells from mouse neural stem cells (NSCs), which served as the somatic cell source. These NSC-derived iPS (NSC-iPS) cells expressing *Oct4*-GFP proliferated for at least for 20 passages. Feeder-free NSC-iPS cells showed expression of pluripotency-related genes, pluripotential epigenetic state, differentiation potential into all three germ layers *in vitro*, and formation of germline chimeras. Our finding demonstrates that NSCs can be reprogrammed into germline-competent iPS cells under feeder-free conditions.

## Materials and Methods

### NSCs culture

We used mouse neural stem cells (NSCs) as a somatic cells for reprogramming. NSCs were derived from OG2/ROSA26 heterozygous double transgenic mice, which were generated by crossing ROSA26 (carrying *neo*/*lacZ* transgene) strain with OG2 transgenic strain (carrying GFP under the control of the *Oct4* promoter, Oct4-GFP) over several generations. Brain tissue was collected from 12.5- to 16.5-day post coitum (dpc) fetuses, which were OG2/ROSA26 heterozygous double transgenic. Neurospheres cultured from the brain tissues were prepared as described in detail in the previous report [25]. The cortex was dissected from the rest of the brain of each mouse and enzymatically dissociated in HBSS (with 2 mM glucose) containing 0.7 mg/ml hyaluronic acid, 0.2 mg/ml kynurenic acid, and 1.33 mg/ml trypsin at 37°C for 30 min. The dissociated cells were passed through a 70-mm nylon mesh (Falcon) to remove large cell clusters. The cells were then centrifuged at 200 g for 5 min and collected by centrifugation in 0.9 M sucrose in 0.5X HBSS at 750 g for 10 min. The cell pellet was resuspended in 2 ml of culture medium, placed on top of 10 ml of 4% bovine serum albumin (BSA) in EBSS solution, and centrifuged at 200 g for 7 min. The culture medium was supplemented with 20 ng/ml epidermal growth factor (EGF; Gibco BRL), 20 ng/ml basic fibroblast growth factor (bFGF), B27 supplement (Gibco BRL), 8 mM HEPES, 2 mM glutamine, 100 U/ml penicillin, and 100 mg/ml streptomycin in DMEM-F12 medium (Gibco BRL). Primary neurospheres were replated onto gelatinized dishes in NSC expansion medium: NS-A media (Euroclone) supplemented with N2 supplement, 10 ng/ml of EGF, and bFGF (Invitrogen), 50 µg/ml bovine serum albumin (BSA Fraction V; Gibco BRL), 1X penicillin/streptomycin/glutamine, and 1X nonessential amino acids (Gibco BRL). Outgrowing cells were trypsinized, replated, and cultured in NSC expansion medium. NSCs were established by dissociation and replated onto gelatin-coated dishes in NSC expansion medium.

### Generation of iPS cells from NSCs

pMX-based retroviral vectors encoding the mouse cDNAs of *Oct4, Sox2, KLf4*, and *c-Myc* [4] were separately co-transfected by packaging defective helper plasmids into 293T cells using Fugene 6 transfection reagent (Roche). 48 hrs post-infection, virus supernatants were collected, filtered, and concentrated as previously described [26]. NSCs (OG2^+/−^/ROSA26^+/−^) were seeded at a density of 1×10^5^ cells per 6-well plate and incubated with virus-containing supernatants of the four factors (1∶1∶1∶1) supplemented with 6 µg/ml protamine sulfate (Sigma) for 24 hrs. On day 7 after viral infection, GFP-positive colonies were transferred onto gelatin coated dishes, and then trypsinized, replated, and cultured in two different types of ES cell media: typical DMEM-based medium supplemented with 15% fetal bovine serum (FBS), 1X penicillin/streptomycin/glutamine, 0.1 mM nonessential amino acids, 1 mM β-mercaptoethanol (Gibco BRL), and 10^3^ units/ml leukemia inhibitor factor (LIF; ESGRO, Chemicon International); and feeder-free GMEM-based medium supplemented with 15% FBS, 1X penicillin/streptomycin/glutamine, 0.1 mM nonessential amino acids, 1 mM β-mercaptoethanol, and 10^3^ units/ml LIF.

### RNA isolation and quantitative RT-PCR

Total RNA was isolated using the RNeasy Mini Kit (QIAGEN) and treated with DNase to remove genomic DNA contamination. 1 µg of total RNA was reverse transcribed with SuperScript III Reverse Transcriptase Kit (Invitrogen) and Oligo(dT) primer (Invitrogen) according to the manufacturer's instructions. Quantitative polymerase chain reaction (PCR) reactions were set up in duplicate with the Power SYBR Green Master Mix (Dakara) and analyzed with the Roche LightCycler 5480 (Roche). Primers used are listed in Table 1.

**Table 1:** Primers and reaction conditions used in qRT-PCR and bisulfite sequencing analysis.

Gene	Tm (°C)	Sequence 5′-3′	Size (bp)	Application	Accenssion No.
*Oct4* (endo)	60	cca atc agc ttg ggc tag ag	129	qRT-PCR	NM_013633
		ctg gga aag gtg tcc ctg ta			
*Sox2* (endo)	60	cac aac tcg gag atc agc aa	190	qRT-PCR	NM_011443
		ctc cgg gaa gcg tgt act ta			
*Nanog* (*endo)*	60	agg ctg att tgg ttg gtg tc	205	qRT-PCR	NM_028016
		ccc agg aag acc cac act cat			
*Oct4* 1st	45	ttt gtt ttt tta ttt att tag ggg g	299	BGS	NM_013633
		atc ccc aat acc tct aaa cct aat c			
*Oct4* 2nd	55	ggg tta gag gtt aag gtt aga ggg	161	BGS	NM_013633
		ccc cca cct aat aaa aat aaa aaa a			
*Nanog* 1st	45	ttt gta ggt ggg att aat tgt gaa	312	BGS	NM_028016
		aaa aaa ttt taa aca aca acc aaa aa			
*Nanog* 2nd	55	ttt gta ggt tgg gat taa ttg tga a	188	BGS	NM_028016
		aaa aaa aca aaa cac caa cca aat			
*Oct4* TG	65	gac ggc atc gca gct tgg ata	320	RT-PCR	N/A
		cca ata cct ctg agc ctg gt			
*Sox2* TG	65	gac ggc atc gca gct tgg ata	442	RT-PCR	N/A
		cgc ttg gcc tcg tcg atg aa			
*Klf4* TG	65	gac ggc atc gca gct tgg ata	308	RT-PCR	N/A
		ggg aag tcg ctt cat gtg ag			
*c-Myc* TG	65	gac ggc atc gca gct tgg ata	399	RT-PCR	N/A
		acc gca aca tag gat gga ga			

qRT-PCR: Quantitative RT-PCR, BGS: Bisulfite genomic sequencing, N/A: Not applicable.

### Bisulfite DNA sequencing analysis

To differentiate the methylated from the unmethylated CG dinucleotides, genomic DNA was treated with sodium bisulfite to convert all unmethylated cytosine residues into uracil residues using EpiTect Bisulfite Kit (QIAGEN) according to the manufacturer's protocol. Briefly, purified genomic DNA (0.5–1 µg) was denaturated at 99°C and then incubated at 60°C. Modified DNA—i.e. after desulfonation, neutralization, and desalting—was diluted with 20 µl of distilled water. Subsequently, bisulfite PCR (BS-PCR) amplification was carried out using 1–2 µl aliquots of modified DNA for each PCR reaction.

PCR amplification for the promoter regions of *Oct4* and *Nanog* was done as previously described [27]. Briefly, the amplified products were verified by electrophoresis on 1% agarose gel. The desired PCR products were used for subcloning using TA cloning vector (pGEM-T Easy Vector; Promega). The reconstructed plasmids were purified, and individual clones were sequenced (Solgent Coporation). Clones with ≥ 90% cytosine conversion were accepted, and all possible clonalities were excluded based on criteria from BiQ Analyzer software (Max Planck Society). Primers used are listed in Table 1.

### In-vitro differentiation of iPS cells and immunofluorescence assay

Differentiation of iPS cells cultured without feeder cells was induced by treatment with retinoic acid (RA). iPS cells were transferred into a suspension culture dish after trypsinization and cultured for 2 days with DMEM (10% FBS) in the absence of LIF. After 2 days, emerging embryoid bodies were treated with 5 µM RA for 8 days in the suspension culture dish and then plated onto a gelatin-coated dish for 10 days. Cells that had differentiated from iPS cells were stained for markers of the three germ layers makers: HNF 3ß for endoderm, Brachyury for mesoderm, and Tuj1 for ectoderm. For immunocytochemistry, cells were fixed with 4% paraformaldehyde for 20 min at room temperature. After washing with PBS, cells were treated with PBS containing 10% normal goat serum and 0.03% Triton X-100 for 45 min at room temperature. Primary antibodies used were anti–ß III tubulin (Tuj1; monoclonal, 1∶1000, Chemicon, Temecula, CA, USA), anti-Brachyury (Brachyury; polyclonal, 1∶1000, Chemicon), and hepatocyte necrosis factor (HNF 3β; monoclonal, 1∶1000, Chemicon). For detection of primary antibodies, fluorescence-labeled (Alexa fluor 488 or 568; Molecular Probes, Eugene, OR, USA) secondary antibodies were used according to the specifications of the manufacturer.

### Aggregation of iPS cells with zona-free embryos

iPS cells were aggregated with denuded post-compacted eight-cell–stage embryos to obtain an aggregate chimera. Eight-cell embryos flushed from 2.5-dpc B6D2F1 female mice were cultured in microdops of embryo culture medium under mineral oil. After cells were trypsinized for 10 seconds, clumps of iPS cells (4–10 cells) were selected and transferred into microdrops containing zona-free eight-cell embryos. Morula-stage embryos aggregated with feeder-free (FF)-iPS cells were cultured overnight at 37 °C, 5% CO_2_. The aggregated blastocysts were transferred into one uterine horn of 2.5-dpc pseudopregnant recipients. Animals were maintained and used for experimentsation under the guidelines of the Institutional Animal Care and Use Committee of Max-Planck Institute for Molecular Biomedicine and CHA University. Institutional review board or ethics committee specifically approved this study (IACUC 090003).

### X-gal staining

For whole fetal embryo staining, collected fetuses were rinsed with PBS and fixed with 4% formaldehyde for 1 hr at 4°C. Fetuses were rinsed three times at room temperature in PBS supplemented with 5 mM EGTA, 0.01% deoxycholate, 0.02% NP40, and 2 mM MgCl_2_. The specimens were washed with PBS and then stained in X-gal staining solution: PBS supplemented with 1 mg/ml 5-bromo-4-chloro-3-indolyl-galactosidase (X-gal; Promega, Madison, WI, USA), 5 mM K_2_Fe(CN)_6_, 5 mM K_4_Fe(CN)_6_, and 1 mM MgCl_2_. Blue staining was visualized by light microscopy.

## Results

### Generation of iPS cells from NSCs in a feeder-free condition

**Figure 1:**
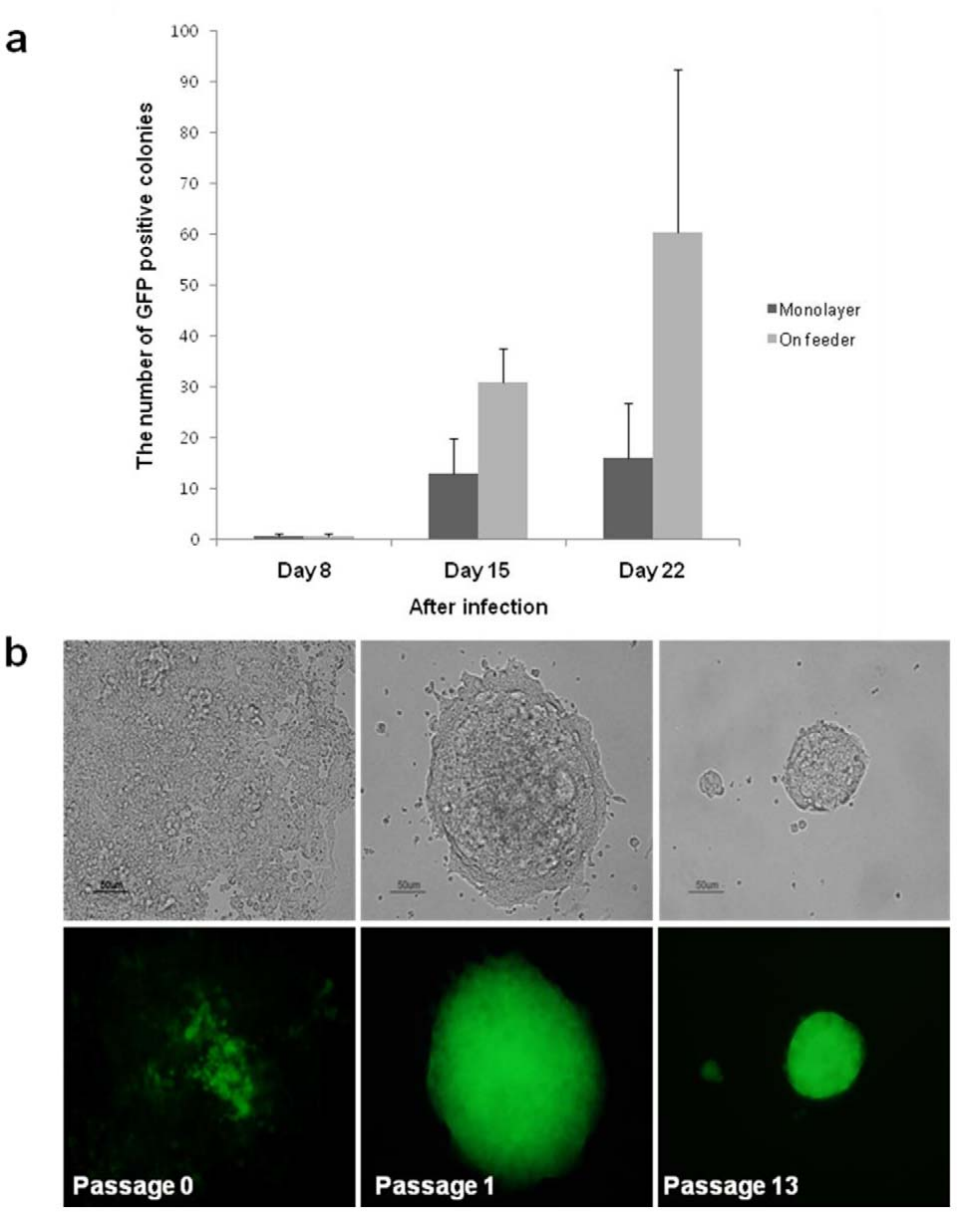
Generation and Cultivation of iPS cells from NSCs Without Feeder Cells. (a) NSCs transduced by four factors were cultured on monolayer without feeder cells or on feeder cells (MEFs), and the number of GFP-positive colonies was counted on days 8, 15, and 22 after viral infection. (b) Phase and fluorescence (GFP) images of FF-iPS cells from NSCs cultured in a feeder-free system with GMEM-based ES cell medium at passages 0, 1, and 13.

We used NSCs obtained from OG2/ROSA26 heterozygous male brain tissue as donor cells for reprogramming experiments. Therefore, these NSCs contain *Oct4*-GFP and *neo*/*LacZ* transgenes. The *Oct4* gene is expressed in pluripotent cells of the mouse embryo up to the gastrulation stage [27]–[29] and is restricted to germ cells thereafter [30]. *Oct4*, which is inactive in NSCs, becomes reactivated when NSCs are reprogrammed into pluripotent cells [25]. We first tested whether NSCs could achieve a state of pluripotency without feeder cells in the culture. NSCs were transduced by four transcription factors encoding *Oct4*, *Sox2*, *Klf4*, and c-*Myc* and cultured on feeder cells (MEFs) or without feeder cells. *Oct4*-GFP–positive colonies were first detected on day 8 after viral infection both in culture without feeder cells and on feeder cells, indicating that initial reactivation of *Oct4*-GFP of NSCs did not require feeder cells. The number of *Oct4*-GFP–positive colonies from monolayer NSCs or on feeder cells was counted on days 8, 15, and 22 after viral infection. Reprogramming efficiency of NSCs cultured on feeder cells was three times higher than that for NSCs cultured without feeder cells (Fig. 1a). These results suggested that initial reactivation of *Oct4*-GFP of NSCs does not require feeder cells but that feeder cells support maintenance of pluripotency and proliferation of iPS cells.

*Oct4*-GFP–positive cells were collected and transferred onto a gelatin-coated dish and cultured in two different types of ES cell medium: typical medium (DMEM-based) and feeder-free medium (GMEM-based) (Fig. 1b). GFP-positive iPS cells plated onto a gelatin-coated dish formed flat colonies, but ES cells cultured on feeder cells formed dome-like colonies. Feeder-free NSC-iPS cells (FF-iPS cells) maintained pluripotency (judged by *Oct4*-GFP expression) in GMEM-based medium but not in DMEM-based medium. In DMEM-based medium, the number of *Oct4*-GFP–positive cells gradually declined until the cells all disappeared (Figure S1), indicating that iPS cells did not maintain pluripotency but were induced to differentiate in a feeder-free condition. FF-iPS cells cultured in GMEM-based medium expressed *Oct4*-GFP and could proliferate for more than two months (passage 20); in addition, the morphology of FF-iPS cell colonies was similar to that of ES cell colonies cultured on feeder cells (Fig. 1b). Taken together, these results suggest that NSCs can achieve pluripotency by defined factors and the resultant iPS cells can maintain ES cell–like morphology under feeder-free conditions.

### Pluripotency marker gene expression and epigenetic reprogramming in FF-iPS cells

**Figure 2:**
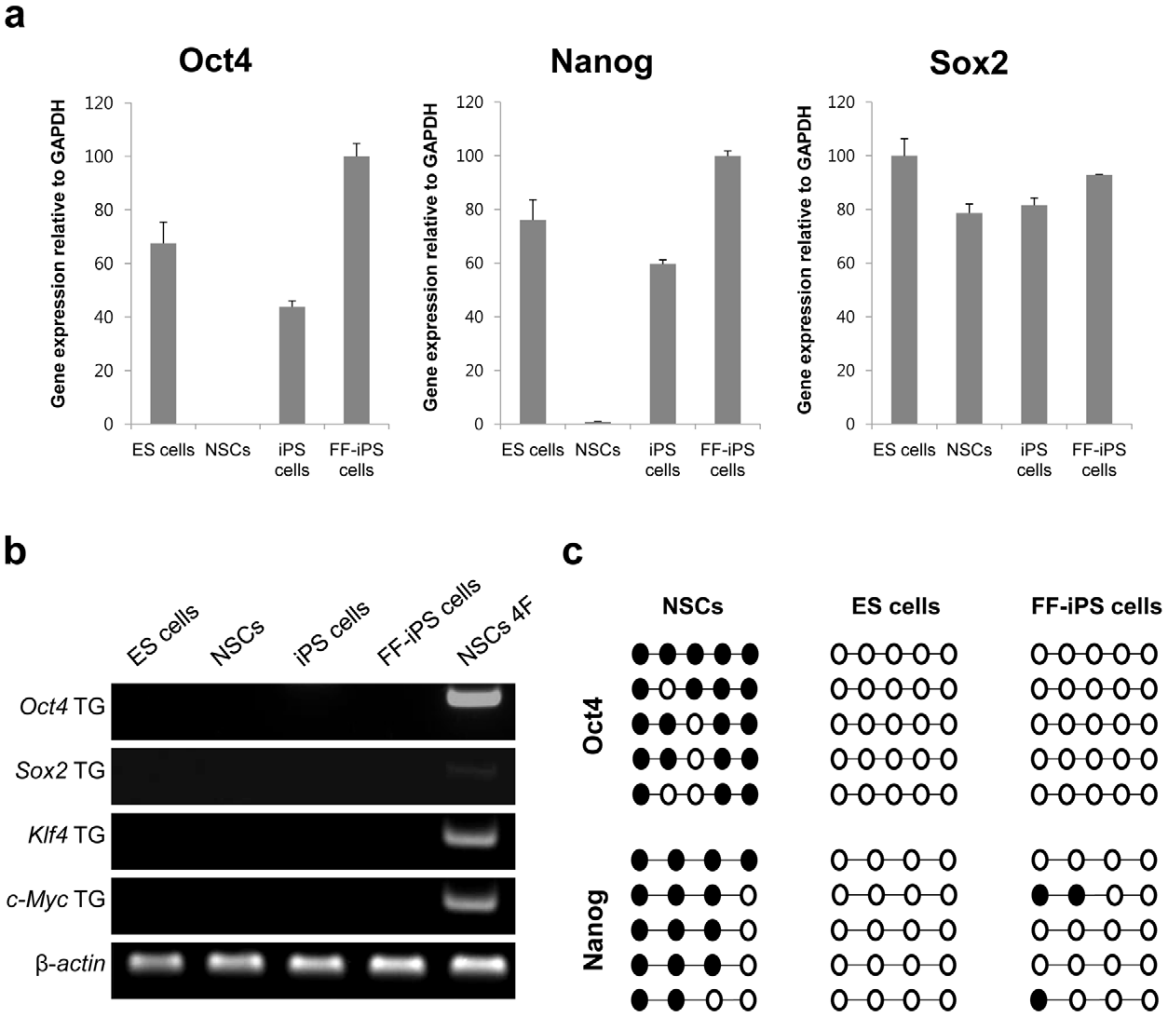
Pluripotency Marker Expression Levels and Methylation Pattern in FF-iPS cells. (a) Expression levels of endogenous factors (*Oct4*, *Sox2*, *Nanog*) quantified by real-time-PCR. (b) RT-PCR analysis for retroviral transgenes. (c) Bisulfite genomic sequencing of the promoter regions of *Oct4* and *Nanog*.

We performed real-time RT-PCR analysis to determine the expression level of pluripotency-related genes, *Oct4* and *Nanog*, in FF-iPS cells (Fig. 2a). FF-iPS cells expressed similar levels of these pluripotency markers to ES and iPS cells, which were cultured on feeder cells. However, retroviral transgenes were silenced in FF-iPS cells (Fig. 2b). Next, we assessed the DNA methylation status of *Oct4* and *Nanog* in FF-iPS cells. Bisulfite genomic sequencing analysis revealed that the promoter regions of these two genes were unmethylated in FF-iPS cells, as shown in pluripotent ES cells (Fig. 2c). However, these regions were hypermethylated in NSCs (Fig. 2c). These results demonstrate that the expression levels and epigenetic status of pluripotency genes in FF-iPS cells are similar to those in ES cells.

### In-vitro and in-vivo differentiation potential of FF-iPS cells

**Figure 3:**
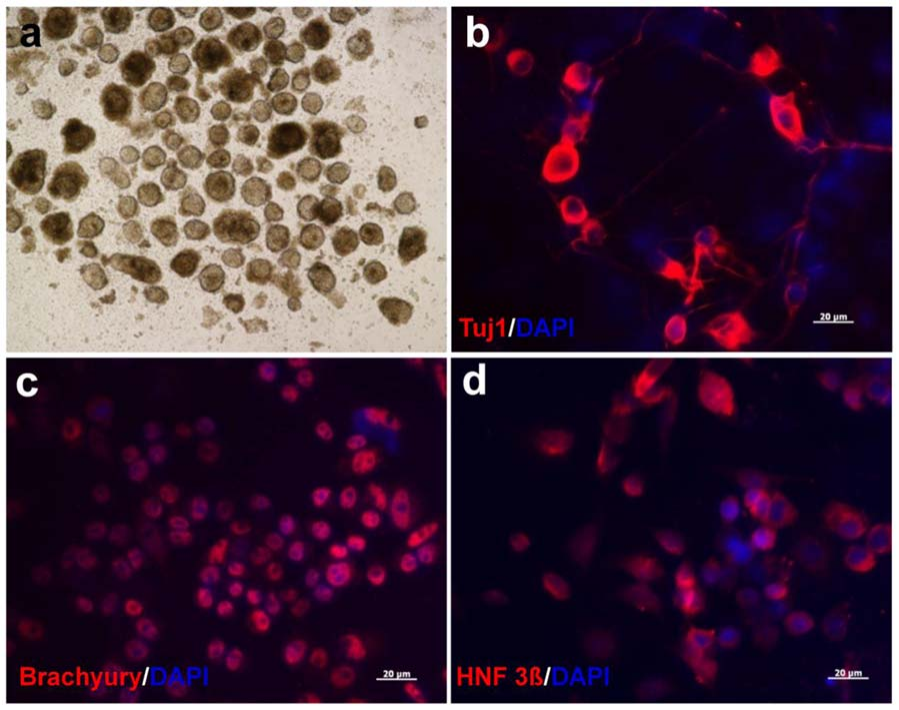
In-vitro Differentiation Potential of FF-iPS Cells. (a) FF-iPS cells could form embryoid bodies (EBs). Immunofluorescence analyses show expression of markers for neuroectoderm (*Tuj1*; red) (b), mesoderm (*Brachyury*; red) (c), endoderm (*HNF 3ß*; red) (d).

**Figure 4:**
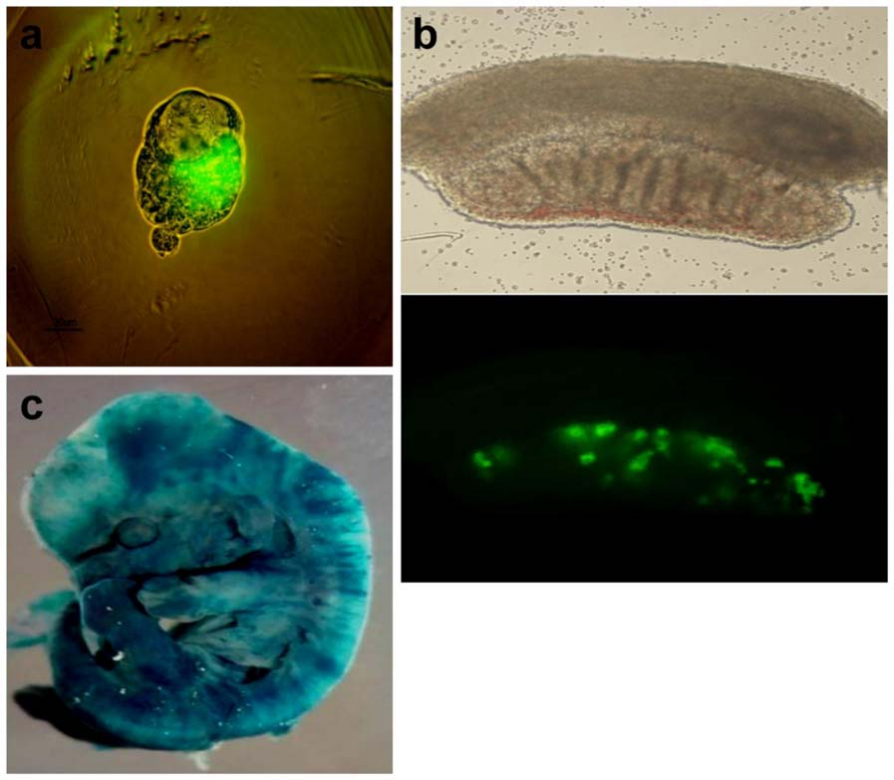
In-vivo Differentiation Potential of FF-iPS Cells. (a) The aggregated embryo shows that FF-iPS cells were incorporated into inner cell mass (ICM) of a normal embryo (*Oct4*-GFP–positive cells in ICM). (b) FF-iPS cells could contribute to germline cell development (*Oct4*-GFP–positive) in a male gonad (13.5 dpc). (c) Chimeric embryo of FF-iPS cells was stained by X-gal.

To investigate the differentiation potential of FF-iPS cells *in vitro*, we determined whether FF-iPS cells could differentiate into three germ layers by embryoid body (EB) differentiation. Well-formed EB structures were observed on day 8 of culture of FF-iPS cells in the absence of LIF (Fig. 3a). These EBs were incubated on a gelatin-coated tissue plate and cultured in MEF medium for 10–15 days. Immunocytochemical analysis showed that FF-iPS cells had differentiated into all three germ layers tissues: ectodermal (Tuj1), mesodermal (Brachyury), and endodermal lineages (HNF 3ß) (Fig. 3b–d). To investigate the *in-vivo* developmental differentiation potential of FF-iPS cells, we performed aggregation analysis. FF-iPS cells had incorporated into the ICM of a normal embryo after aggregation with zona-free morula embryos (Fig. 4a). When the blastocysts were transferred into the uterus of pseudopregnant mice, the FF-iPS cells formed chimeric embryos (13.5 dpc), showing germline contribution (Fig. 4b–c). Taken together, these results demonstrate that FF-iPS cells possess pluripotent differentiation and developmental potential *in vitro* and *in vivo*.

## Discussion

In the present study, we showed that iPS cells can be induced from NSCs by defined transcription factors and maintained in a feeder-free condition. Feeder-free NSC-iPS cells proliferated for at least for 20 passages, maintaining a cellular state of pluripotency and differentiation potential into all three germ layers *in vitro* and *in vivo*. These FF-iPS cells expressed similar levels of pluripotency-related genes to ES cells and the promoter regions of *Oct4* and *Nanog* were hypomethylated in FF-iPS cells. FF-iPS cells could form EBs, which could differentiate into derivates of the three embryonic germ layers. Moreover, FF-iPS cells have passed the stringent test for pluripotency by generating germline chimeras.

Chen et al. suggested that the efficiency of iPS cell generation without feeder cells was not different from that of reprogramming on feeder cells [20]. However, in this study, we showed that efficiency of reprogramming induced on feeder cells was three times higher than that without feeder cells (Fig. 1a), indicating that feeder cells support self-renewal and pluripotency of early-stage reprogrammed cells. Discrepancy in these results may be due to the types of somatic cells that are used for reprogramming. In using fibroblasts as donor cells, individual fibroblasts that had failed to reprogram could function as feeder cells and support those fibroblast cells that had been successfully reprogrammed. By using NSCs as donor cells, we could eliminate the possibility of any support from donor cells, as ES cells display a tendency to differentiate (data not shown), thus indicating that NSCs do not function as feeder cells.

NSCs are efficiently reprogrammed into a pluripotent state in a nuclear reprogramming system, such as cell-cell fusion and direct reprogramming [31], [32]. The fact that NSCs which already express *Sox2* and *c-Myc* could acquire pluripotency by only one factor, *Oct4*, suggests that NSCs may exist in an intermediate state between differentiated and pluripotent stem cells [33]. In this study, *Oct4*-GFP was detected on day 7 after retroviral infection (*Oct4*, *Sox2*, *Klf4*, and *c-Myc*) without feeder cells. NSCs achieved pluripotency (judged by activation of *Oct4*-GFP) as early as the control group (on day 8 after infection) when cultured on feeder cells, indicating that the feeder cell is not a crucial factor in the initial step/stages of reprogramming. However, in a drug-inducible system, neural stem/progenitor cells could be reprogrammed only with feeder cells [19]. Although there is no clear explanation for this, feeder dependency may be affected not only by the somatic cell type, but also by the reprogramming-inducing method.

We demonstrated that iPS cells reprogrammed from NSCs could maintain pluripotency in a feeder-free system. In typical DMEM-based medium, the *Oct4*-GFP–positive cells gradually disappeared, as they differentiated. However, previous reports have shown that conditioned medium containing factors secreted by MEFs [16], [34] could support maintenance of pluripotency [35]–[38]. So we tested whether MEF-conditioned medium supports pluripotency maintenance of iPS cells in a feeder-free culture system. When the FF-iPS cells were cultured in a mixture of DMEM-based ES cell medium and MEF-conditioned medium (1∶1), *Oct4*-GFP–positive cells disappeared as early as those in typical DMEM-based ES cell medium. Mouse ES cells are generally cultured in DMEM-based medium with feeder cells, in which mouse ES cells form domed colonies. Mouse ES cells also can be cultured in GMEM-based ES medium without feeder cells, in which ES cells form flat colonies. However, GMEM-based medium is used only for specific ES cell line, such as HM-1 and nanog overexpressing cell line [32], [39]. Interestingly, we found that NSC derived iPS cells maintained *Oct4*-GFP expression for more than two months (passage 20) and form domed colonies in GMEM-based ES medium without feeder cells. Collectively, these data indicate that GMEM-based medium is more effective for cultivation of mouse iPS cells in a feeder-free system than typical DMEM-based medium or MEF-conditioned medium.

It has been suggested that in feeder-free system, the pluripotency of ES and iPS cells could be maintained in conditioned-medium [40] or by chemical support [35], [41]. Our study demonstrated that NSCs, which do not function as a feeder cells, were successfully reprogrammed into a pluripotent state and could maintain pluripotency without feeder cells in a simple GMEM-based medium, indicating that factors secreted by feeder cells are not essential in the initial/early stages of reprogramming and for pluripotency maintenance.

## Supporting Information

Figure S1 **Spontaneous Differentiation of FF-iPS Cells in Typical ES Cell Medium.** Phase contrast and fluorescence (GFP) images of FF-iPS from NSCs differentiated in a feeder-free system with typical ES cell medium at passages 1, 3, and 13.
(TIF)
