# Supplementary figures and images for: Neural Stem Cells Achieve and Maintain Pluripotency without Feeder Cells
Source: PLoS One. 2011 Jun 24;6(6):e21367. doi: 10.1371/journal.pone.0021367 (PMC3123318; doi:10.1371/journal.pone.0021367)

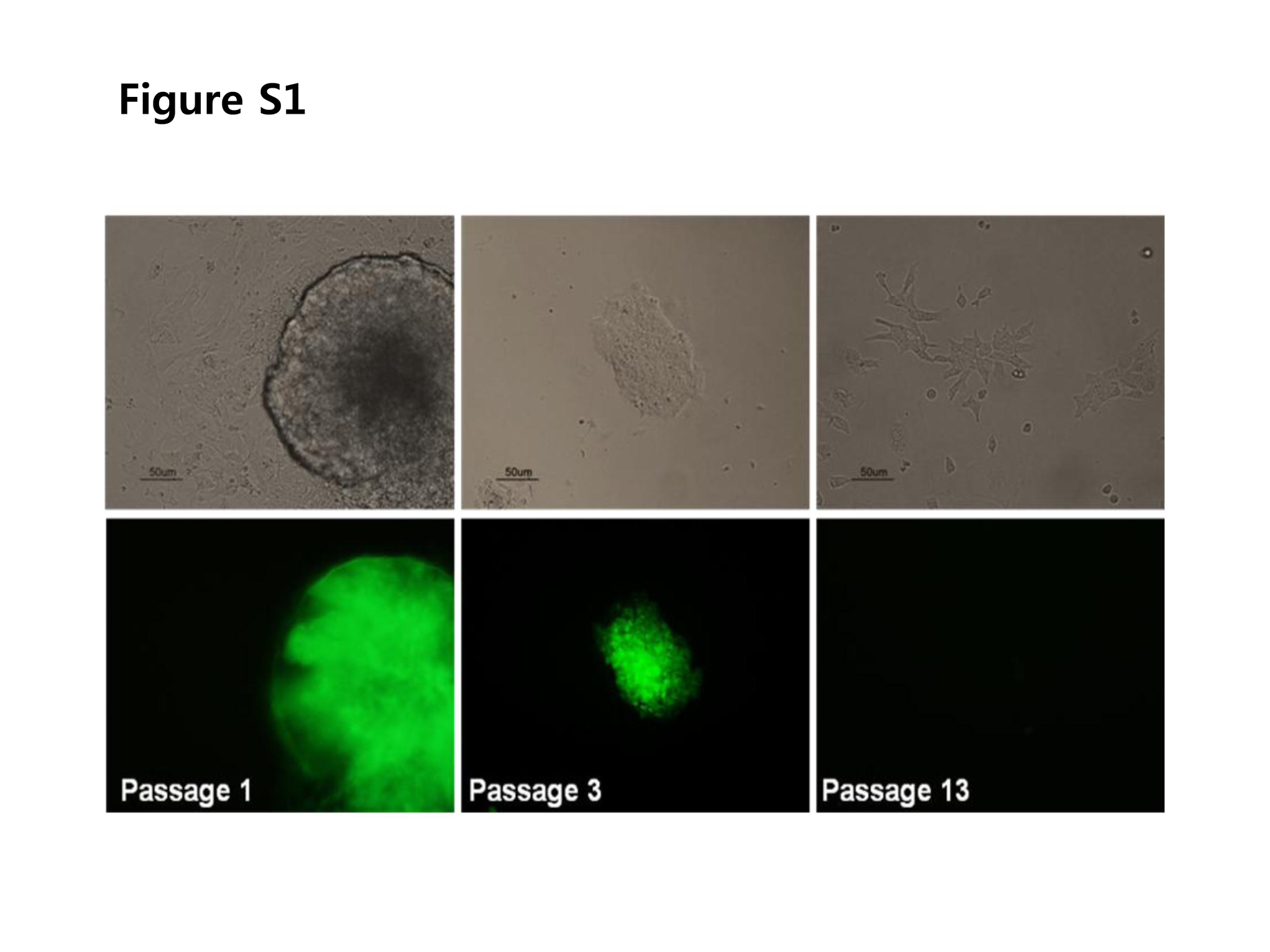

Supplement: Figure S1 — Spontaneous Differentiation of FF-iPS Cells in Typical ES Cell Medium. Phase contrast and fluorescence (GFP) images of FF-iPS from NSCs differentiated in a feeder-free system with typical ES cell medium at passages 1, 3, and 13. (TIF) [file pone.0021367.s001.tif]
